# The bacterial protein CNF1 as a new strategy against *Plasmodium falciparum* cytoadherence

**DOI:** 10.1371/journal.pone.0213529

**Published:** 2019-03-07

**Authors:** Valeria Messina, Stefano Loizzo, Sara Travaglione, Lucia Bertuccini, Maria Condello, Fabiana Superti, Marco Guidotti, Pietro Alano, Francesco Silvestrini, Carla Fiorentini

**Affiliations:** 1 Department of Infectious Diseases, Istituto Superiore di Sanità, Rome, Italy; 2 Italian Center for Global Health, Istituto Superiore di Sanità, Rome, Italy; 3 Core Facilities, Istituto Superiore di Sanità, Rome, Italy; 4 National Center for Drug Research and Evaluation, Istituto Superiore di Sanità, Rome, Italy; 5 National Center for Innovative Technologies in Public Health, Istituto Superiore di Sanità, Rome, Italy; 6 Department of Food Safety, Nutrition and Veterinary Public Health, Istituto Superiore di Sanità, Rome, Italy; Liverpool School of Tropical Medicine, UNITED KINGDOM

## Abstract

*Plasmodium falciparum* severe malaria causes more than 400,000 deaths every year. One feature of *P*. *falciparum*-parasitized erythrocytes (pRBC) leading to cerebral malaria (CM), the most dangerous form of severe malaria, is cytoadherence to endothelium and blockage of the brain microvasculature. Preventing ligand-receptor interactions involved in this process could inhibit pRBC sequestration and insurgence of severe disease whilst reversing existing cytoadherence could be a saving life adjunct therapy. Increasing evidence indicate the endothelial Rho signaling as a crucial player in malaria parasite cytoadherence. Therefore, we have used the cytotoxic necrotizing factor 1 (CNF1), an *Escherichia coli* protein able to modulate the activity of Cdc42, Rac, and Rho, three subfamilies of the Rho GTPases family, to study interactions between infected erythrocytes and cerebral endothelium in co-culture models. The main results are that CNF1 not only prevents cytoadherence but, more importantly, induces the detachment of pRBCs from endothelia monolayers. We first observed that CNF1 does affect neither parasite growth, nor the morphology and concentration of knobs that characterize the parasitized erythrocyte surface, as viewed by scanning electron microscopy. On the other hand, flow cytometry experiments show that cytoadherence reversion induced by CNF1 occurs in parallel with a decreased ICAM-1 receptor expression on the cell surface, suggesting the involvement of a toxin-promoted endocytic activity in such a response. Furthermore, since the endothelial barrier functionality is compromised by *P*. *falciparum*, we conducted a permeability assay on endothelial cells, revealing the CNF1 capacity to restore the brain endothelial barrier integrity. Then, using pull-down assays and inhibitory studies, we demonstrated, for the first time, that CNF1 is able not only to prevent but also to cause the parasite detachment by simultaneously activating Rho, Rac and Cdc42 in endothelial cells. All in all our findings indicate that CNF1 may represent a potential novel therapeutic strategy for preventing neurological complications of CM.

## Introduction

*Plasmodium falciparum* malaria is a leading cause of ill health, neuro-disability and death in tropical countries [1]. Every year, there are over 500 million clinical cases, with one percent of symptomatic infections that may become complicated and develop into severe malaria. Severe *falciparum* malaria encompasses a broad range of disease manifestations, including cerebral malaria (CM) [2]. Although the CM mechanisms leading to death are still debated [3], CM pathology starts from sequestration of infected and non-infected red blood cells in the venules and capillaries of the brain, a process called ‘cytoadherence’ [4], with the consequent formation of microvascular obstruction that can lead to hypoxia and inadequate tissue perfusion [5]. Cytoadherence needs the formation on the surface of parasitized red blood cells (pRBCs) of protrusions named knobs, which bind to several endothelial adhesion molecules variably expressed in different organs, forming a physical ‘engagement’ of pRBCs with endothelial cells [6]. It has been hypothesized that pRBC adhesion to endothelial cells directly triggers the Rho signaling activation in the host cells [7]. This is supported by the fact that members of the Rho family of small GTPases, which are activated and inactivated by binding to GTP and GDP, respectively, are the first intermediates of the intracellular signaling mediating the engagement of various receptors, including ICAM-1, VCAM-1 and selectins, thus playing a pivotal signaling role in cytoadherence [8]. The Rho GTPases are also involved, albeit indirectly as effectors, in the pathways governed by the Endothelial cell protein C receptor (EPCR), an additional important actor in this scenario [9,10].

Treatments for CM, all of which target the parasite, are at present insufficient and other approaches are needed to prevent the deadly outcome of CM. Improvements in early diagnosis of CM [11] show that a timely treatment could be effective. It would be also highly desirable to develop a prophylaxis able to prevent cytoadhesion. This therapeutical approach is fortified by the results obtained using compounds that act on the adherence between the host cell and pRBCs. In fact, some of these compounds have been screened for their potential to inhibit cytoadhesion [8,12–14]. Recently, it has been shown that anti-ICAM-1 and anti-CD36 monoclonal antibodies are able to inhibit as well as to reverse binding of *P*. *falciparum* laboratory strains and patient isolates to endothelial cells *in vitro*, providing the proof of concept that established ligand–receptor interactions can be reversed and that this has a potential therapeutic value for CM [15]. However, the physiological major role of ICAM-1 and CD36 in leukocyte trafficking and immune response raises the reasonable concern that heavily blocking, by a monoclonal antibody therapy, the normal function of these receptors may seriously affect the host [16]. On the other hand, other Authors demonstrated the feasibility of this approach. In fact, heparin-derived drugs, such as glycosaminoglycan, are able to both prevent and reverse cytoadherence [14,17]. In particular, Sevuparin has recently been evaluated in patients suffering for uncomplicated falciparum malaria, with encouraging results [18]. Thus, these recent findings substantiate the validity of developing anti-adhesion therapies as potential life-saving approaches against fatal CM.

The Escherichia coli cytotoxic necrotizing factor 1 (CNF1), a protein toxin that specifically modulates the family of Rho GTPases (including Rho, Rac and Cdc42 subfamilies) [19–21] and reorganizes the cell cytoskeleton, may represent a new strategy to counteract parasite cytoadherence. Rho GTPases are constitutively activated by CNF1 through deamidation of a critical glutamine residue that locks them in their activated, GTP-bound state. The threshold of this activation is afterward attenuated since cells, detecting the high levels of activated Rho GTPases, trigger their ubiquitination and degradation to more physiological levels. CNF1 can modulate ICAM-1 expression in epithelial cells [22] and in natural killer (NK) cells [23] by acting on the cytoskeleton. Also, CNF1 protects epithelial cells against apoptosis [22], reinforces NK cells activity [23] and brings back the expression of certain neuroinflammatory markers to control levels in pathological animal models, with a rescue of the pathological phenotypes [24,25]. All this renders CNF1 a suitable candidate to affect host cell pathways involved in *P*. *falciparum* cytoadherence in view of its potential use as a therapeutic tool against CM.

The aim of this study was to evaluate if CNF1, by modulating the Rho GTPases pathway and the host cell cytoskeleton, can interfere with the mechanism necessary for pRBC adhesion to host endothelial cells, by preventing adhesion or promoting the detachment of pRBCs as well as restoring the endothelial barrier integrity.

## Materials and methods

### CNF1 preparation

CNF1 was obtained from the 392 ISS strain (kindly provided by V. Falbo, Istituto Superiore di Sanità, Rome, Italy) and purified as previously described [26]. The recombinant protein CNF1 C866S, in which the enzymatic activity on Rho GTPases is abrogated by change of cysteine with serine at position 866 [21], was purified as previously described [26] and used as a negative control in post-treatment. The plasmid coding for CNF1 C866S was kindly provided by E. Lemichez (U627 INSERM, Nice, France). For experiments, a concentration of 10^−10^ M for both CNF1 and CNF1 C866S was used.

### Parasite culture

The *P*. *falciparum* ItG line [27] and AQ104 [28] were cultured in human 0+ erythrocytes at 5% hematocrit under 5% CO_2_, 2% O_2_, 93% N_2_. Cultures were grown in medium containing RPMI 1640 medium (Gibco, Carlsbad, CA, USA) supplemented with 25 mM Hepes, 50 μg/mL hypoxanthine, 0.25 mM NaHCO_3_, 50 μg/mL gentamicin sulfate, and 10% pooled heat-inactivated 0+ human serum. For parasite synchronization, cultures at 5–8% parasitaemia at 5% haematocrit were centrifuged at 2,000 rpm for 10 min through a 60% Percoll cushion and slow sedimenting schizonts used to static cell assay. Prior to use, parasites were washed twice in binding buffer (RPMI 1640 medium, supplemented with 6 mM glucose, pH 7.2) and re-suspended at 1% haematocrit and 3% parasitaemia (Giemsa staining).

### Endothelial cells

HBMEC-60 bone marrow endothelial cells [29] and HBEC-5i cerebral microvascular endothelium cells (ATCC CRL3245, Manassas, VA, USA) were routinely grown in 1% gelatin-coated culture flasks using DMEM:F12 with FBS 10%.

### *In vitro P*. *falciparum* drug susceptibility assay

CNF1 was diluted with medium to achieve the required concentrations. Compounds were placed in 96-well plates (Euroclone, Milan, Italy) and serial dilutions were made in a final volume of 100 μl/well. Asexual parasite stages derived from asynchronous cultures with parasitaemia of 1–1.5% were distributed into the plates (100 μl/well, final hematocrit 1%) and incubated for 48 h at 37°C. Epoxomicin (Sigma, St Louis, MO, USA) was used as reference control for the asexual stage [30]. Three experiments in duplicate were performed with asexual parasites. Parasite growth was determined spectrophotometrically by measuring the activity of the parasite lactate dehydrogenase (pLDH), according to a modified version of Makler’s method in control and treated cultures [31]. Briefly, the drug treated culture was resuspended and 20μl/well were transferred in a plate containing 100 μl Malstat reagent [0.11% v/v Triton-100; 115.7 mM 123 lithium L-lactate; 30.27 mM Tris; 0.62 mM 3-acetylpyridine adenine dinucleotide (APAD; Sigma- 124 Aldrich); adjusted to pH 9 with 1 M HCl and 25 μl PES/NBT (1.96 mM nitro blue tetrazolium 125 chloride; 0.24 mM phenazine ethosulphate) to perform the pLDH assay. The plate was read at the wavelength 650nm using a microplate reader Synergy4 (BioTek, Winooski, VT, USA) and the results expressed as 50% inhibitory concentrations (IC50).

### Scanning electron microscopy analysis

All samples were processed for scanning electron microscopy (SEM), as described [32]. Purified *P*. *falciparum* trophozoites treated or not with CNF1 were fixed with 2.5 glutaraldehyde in 0.1M sodium cacodylate buffer (pH 7.4) and were let to adhere on poly-lysine-coated glass coverslips for 4 h at room temperature. The HBEC5i cell monolayers, after treatments with CNF1 and Cdc42 inhibitor ML141, were fixed with 2.5 glutaraldehyde in 0.1M sodium cacodylate buffer (pH 7.4) over night at 4°C. Then both kind of samples were washed in cacodylate buffer and post-fixed with 1% OsO4 in 0.1M sodium cacodylate buffer for an additional 1 h at room temperature. Samples were rinsed and dehydrated through a graded series of ethanol solutions (30–100% ethanol), critical point dried and gold sputtered (thickness 30 nm), and examined by FEG Scanning Electron Microscope (Inspect F–FEI). The number of knobs induced by the *P*. *falciparum* trophozoites on the red cell surface were analyzed on high magnification SEM images by IMAGE J [33] and expressed as density of knobs per unit area (knobs/μm^2^). For each sample of mature trophozoites, treated with CNF1 or not, ten slightly flat areas of trophozoite images were chosen for the analysis and the number of knobs, recorded by IMAGE J elaboration (S1 Fig), were related with the area analyzed to obtain the knobs/μm^2^ values.

### Protein extraction and western blot

Cells were lysed in boiled Sample Buffer 1x (50 mM Tris-HCl pH 6.8, 2% SDS, 10% glycerol, 100 mM DTT). In total, 30 μg of total protein extracts were resolved on 8% SDS-PAGE and electrically transferred onto poly(vinylidene difluoride) membranes (PVDF, Bio-Rad Laboratories, Hercules, CA, USA) membranes. Membranes were blocked with TBS-T (20 mM Tris-HCl pH 7.4, 150 mM NaCl, 0.02% Tween-20) containing 5% skimmed milk (Bio-Rad), for 1 h at room temperature, and then incubated overnight at 4°C with primary antibodies diluted in TBS-T containing 5% milk. The following antibodies were used: mouse monoclonal anti-ICAM-1 (Santa Cruz Biotechnology, Dallas, TX, USA; working dilution 1:500), rabbit polyclonal anti-CD36 (Santa Cruz Biotechnology, working dilution 1:500), mouse monoclonal anti-α-tubulin (Sigma-Aldrich, working dilution 1:10,000). After extensive washing, immunecomplexes were detected with horse-radish peroxidase conjugated species-specific secondary antibodies (Jackson Laboratory, Bar Harbor, ME, USA) followed by enhanced chemiluminescence reaction (Millipore Corporation, Billerica, MA, USA). Proteins detected by immunoblotting were quantified by densitometry (ChemiDoc imaging system, BioRad) and normalized as a function of α-tubulin with Image-Lab 5.0 software (Bio-Rad).

### Fuorescence microscopy

HBEC-5i cells, seeded on glass coverslips, were fixed in 4% paraformaldehyde in phosphate-buffered saline (PBS) and permeabilized with Triton X-100 (0.2%, Sigma-Aldrich). For F-actin detection, cells were stained with FITC (fluoresceine isothyocianate)-phalloidin (Sigma-Aldrich; working dilution 0.5 μg/ mL in PBS) for 30 min at 37°C. Nuclei were stained with Hoechst 33258 (Sigma-Aldrich). Finally, following extensive washes, samples were mounted on glass coverslips and observed with an Olympus BX51 fluorescence microscope (Tokyo, Japan).

Phalloidin immunofluorescence intensity was analyzed by IMAGE J [33], in order to quantify the F-actin polimeryzation induced by CNF1 on endothelial cells. Statistical analysis was performed on five images for each sample (n = 5), acquired at the same magnification, fluorescence exciting and gain conditions. The color channels were split and only the green channel was analyzed for each image. Furthermore, areas with the highest concentration of cells were selected in order to have the best cell/background ratio. Quantification of phalloidin intensity signal derives from the means of grayscale intensity value for each image.

### Cell viability assay

Effects of CNF1 and of the mutant CNF1 C866S on HBEC-5i endothelial cell viability were evaluated by mean of the neutral red uptake assay. Briefly,1x10^4^ cells/well were seeded onto 96-well plates (Euroclone). After 24 h, cells were treated with CNF1 or CNF1 C866S at the following dilutions: 1.25 x 10^−8^ M, 2.5 x 10^−9^ M, 5.0 x 10^−10^ M, 1.0 x 10^−10^ M, in culture medium (DMEM:F12 with FBS 10%), for 4 and 24 h. At each time point, plates were incubated for 3 h with complete medium containing 50 μg/mL neutral red. Cells were subsequently washed in PBS and the dye extracted in each well with 1% glacial acetic acid in 50% ethanol. The uptake was quantified by reading the absorbance with an ELISA reader (Packard Fusion Microplate Reader) at 540 nm.

### ICAM-1 surface expression

To evaluate ICAM-1 surface expression, cell suspensions were incubated in phosphate-buffered saline (PBS) solution with 1% bovine serum albumin (BSA), 10% fetal bovine serum (FBS), and 10% human serum AB to saturate aspecific sites. Then, cells were incubated with ICAM-1 mouse monoclonal antibody (1 μg per 1x10^6^ cells) (Santa Cruz Biotechnology, INC, Dallas, Texas, USA) for 30 min at 4°C, washed with cold PBS, and then incubated with secondary Alexa 488 goat anti-mouse IgG (1:100; Molecular Probes, USA) antibody for 30 min at 4°C. For isotypic control, cells were labeled with IgG2a (Sigma Chemical Co., St Louis, MO, USA) (1 μg per 1x10^6^ cells). Dead cells were excluded from the analysis by staining with trypan blue solution (Sigma). After washing with PBS, flow cytometric analyses were carried out through BD LSR II flow cytometer (Becton, Dickinson &Company, Franklin Lakes, NJ, USA) equipped with a 15 mW, 488 nm, air-cooled argon ion laser and a Kimmon HeCd 325 nm laser. At least 10,000 events were acquired in log mode.

For the quantitative evaluation of ICAM-1 expression, the mean fluorescence channel (MFC) for each sample was calculated by FACS Diva Software (Becton, Dickinson & Company). In the figure, arbitrary units were calculated by the formula: MFC (sample labeled with ICAM-1 antibody)/ MFC (sample labeled with IgG2a).

### Permeability assay

Permeability of endothelial monolayers cultured on Transwell filters was assayed using fluorescein isothiocyanate–dextran FD4 (average mol wt 3,000–5,000; Sigma-Aldrich). 30,000 endothelial cells were seeded on transwell. For pre-treatment assays, monolayers were incubated with CNF1 for 4 h, then ItG schizonts (1% parasitaemia, 0,5% haematocrit) were added to the top of the transwell and after 2 h the upper and lower compartments were washed with Hepes medium, followed by the addition of the FITC-Dextran (10 μg/ml) to the upper compartment. Uninfected RBCs and tumor necrosis factor (TNF)-α (10 ng/mL) were used as negative and positive controls. For post treatment assays, transwells were washed with incomplete medium after parasites adhesion and exposed to CNF1 for 2 h. After incubation, 100 μl of medium from the lower compartment was collected and fluorescence was measured in a spectrofluorimeter (λEX 485 nm; λEM 525 nm).

### Pull-down of activated Rho, Rac and Cdc42 GTPases

Pull-down assay was performed as previously described [34]. Cells were lysed in 1) 50 mM HEPES, pH 7.4, 0.5% sodium deoxycholate, 1% NP-40, 0.1% SDS, 0.5 M NaCl, and 10 mM MgCl_2_, *plus* protease inhibitors (to detect Rho-GTP) or 2) 50 mM HEPES, pH 7.4, 0.1 M NaCl, 10 mM MgCl_2_, 5% glycerol, 1% NP-40, and 10 mM NaF, *plus* protease inhibitors (to detect Rac/Cdc42-GTP). The cleared lysates were incubated with 80 μg of glutathione S-transferase (GST)-Rhotekin (for Rho; Millipore) and GST-PAK-CD (for Rac/Cdc42, prepared as described previously [34]) fusion proteins, bound to glutathione-coupled Sepharose beads (GE Healthcare, Amersham, UK) for 40 min at 4°C. Beads were washed three times in 1) 50 mM MgCl_2_, plus protease inhibitors (for Rho) or 2) 50 mM HEPES, pH 7.4, 0.1 M NaCl, 10 mM MgCl_2_, 5% glycerol, 0.5% NP-40, and 10 mM NaF, plus protease inhibitors (for Rac/Cdc42). The bound proteins were eluted in Sample buffer before being subjected to SDS-PAGE and immunoblotting with the following antibodies: mouse monoclonal anti-RhoA (Millipore, working dilution 1:500), mouse monoclonal anti-Rac1 (BD Biosciences Transduction Laboratories; San Jose, CA, USA; working dilution 1:3,500), and mouse monoclonal anti-Cdc42 (Santa Cruz Biotechnology, working dilution 1:500), anti-α-tubulin (Sigma-Aldrich, working dilution 1:10,000). Whole-cell lysates (2% of input) were analyzed in parallel. Rho, Rac1 and Cdc42-GTP detected by immunoblotting were quantified by densitometry (ChemiDoc imaging system, BioRad) and normalized as a function of total Rho, Rac and Cdc42 proteins loaded in the assay with Image-Lab 5.0 software (Bio-Rad).

### Static cell adhesion assay

Endothelial cells were seeded onto 1% gelatin coated 13 mm Thermanox coverslips (Nalgene, Nunc, Penfield, NY, USA). Once confluent, they were incubated overnight at 37°C with 1 ng/ml rTNF-α [35] (Invitrogen). For pre-treatment experiments, endothelial cells were incubated with 10^−10^ M CNF1 for 12 h. Cells were washed with DMEM and incubated with 0.5 ml of parasite suspension (3% parasitaemia, 1% haematocrit) for 2 h at 37°C. After washing in RPMI 1640, coverslips were placed upside down in new clean microplate wells filled with fresh binding medium for 30 min. The remaining unbound cells detached from the endothelial layer sinking down by gravity. The procedure was repeated twice. For post-treatment experiments cells were treated with 10^−10^ M CNF1 (or CNF1 C866S) for 4 h, and washed again following the same procedure as above. For RhoA and Rac1/Cdc42-inhibition, post-treatment experiments were performed by adding 10 μM Y27632 (Calbiochem, Minneapolis, MN, USA) or 10 μM ML141 (Calbiochem), respectively, to the culture medium either (i) 1 h before CNF1 treatment or (ii) together with CNF1. Adherent cells were then fixed using 1% glutaraldehyde for 1 h and then stained with 5% Giemsa for 30 min. Coverslips were dried and mounted on slides using DPX mounting buffer (BDH Lab Supplies, UK). For each condition/line bound parasites in triplicate wells were counted at 400× magnification and their numbers expressed as the number of pRBC per mm^2^. All experiments were repeated at least three times.

### Static protein assay

Static protein binding assays were carried out spotting 2μl of ICAM-1-rc (Biolegend, 552904; 25, 50 or 100 μg/ml) onto 60 mm diameter bacteriological Petri dishes and incubated in a humidified chamber for 2 h at 37°C. Proteins were aspirated off and dishes blocked overnight at 4°C in 1% BSA/PBS. Blocking solution was removed, the dish washed in binding buffer and 2 ml of parasite suspension at 3% parasitaemia and 1% haematocrit added to each dish. Dishes were incubated at 37°C for 1 h, with re-suspension for every 10 min. Unbound RBC and pRBC were removed by repeated washing, bound cells fixed with 1% glutaraldehyde for 1 h and then stained with 5% Giemsa for 20 min. Levels of adhesion were quantified microscopically.

### Statistical analysis

Data are presented as mean ± SEM from three independent experiments. The parametric analyses of variance (ANOVA) was used for the analysis of statistical significance. When a significant interaction was detected, we also performed Bonferroni post hoc tests. A p value < 0.05 was considered significant.

## Results and discussion

### CNF1 not only prevents but also reverses the cytoadherence of infected erythrocytes to endothelial cells

**Fig 1:**
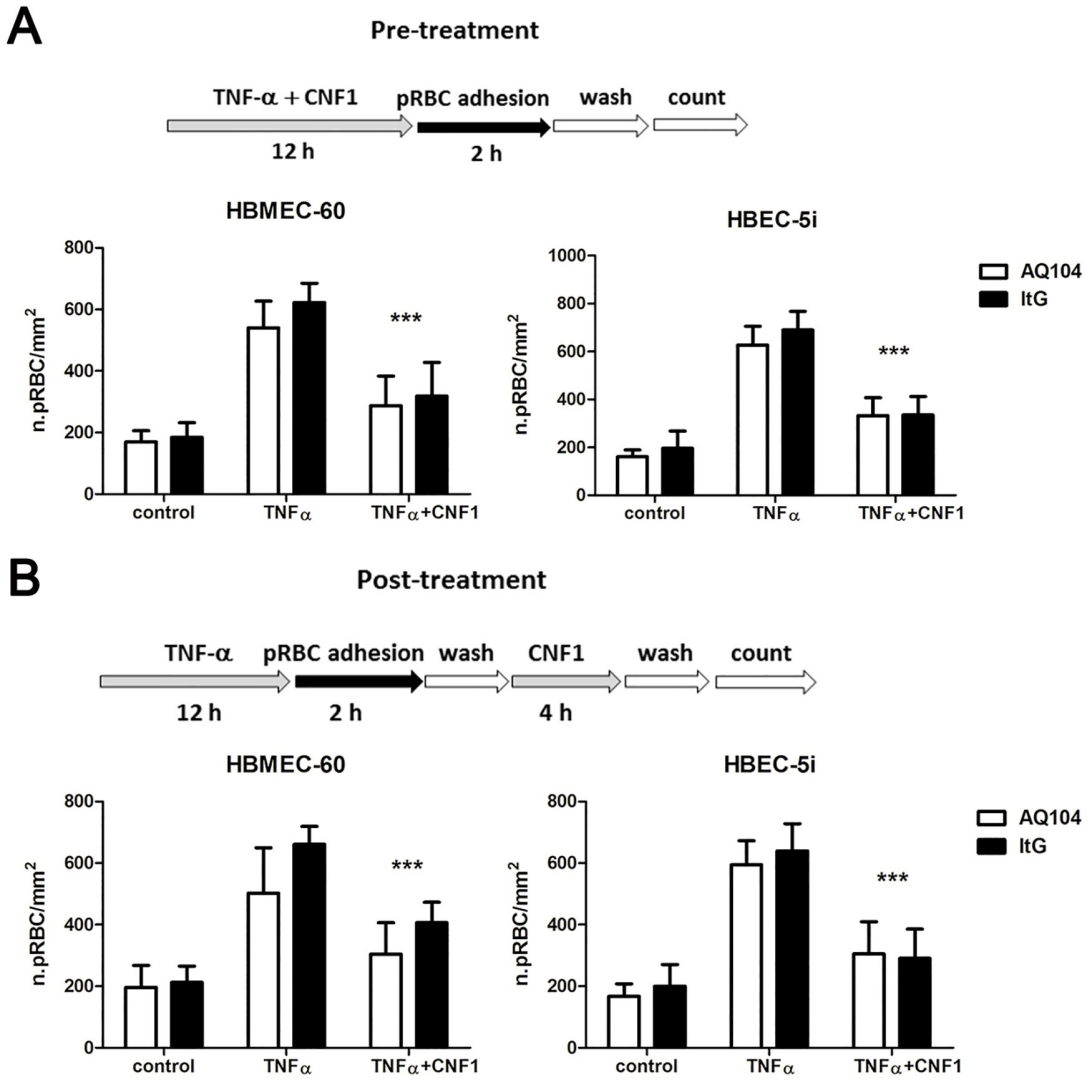
CNF1 decreases *P*. *falciparum* endothelial cytoadherence. HBMEC-60 and HBEC-5i endothelial cells were tested in two different experimental conditions: (A) overnight pre-incubation with CNF1 before the adhesion process of pRBCs (pre-treatment assay) and (B) treatment with CNF1 for 4 h, after the adhesion assay (post-treatment assay). CNF1 was able either to prevent parasite citoadherence (A) or to induce parasite detachment (B), in both cell lines and with both *P*. *falciparum* clones ItG and AQ104. pRBC bound were expressed as raw numbers of pBRC per mm^2^. Data represent mean ± SEM from at least three experiments. *** p<0.001, **p<0.01, *p>0.05.

We first investigated whether CNF1 inhibited the adhesion of two different parasite lines: the asexual stage parasites (trophozoites and schizonts) of the *P*. *falciparum* clone ItG, a reference clone in *in vitro* studies on parasite cytoadhesion, and a recently adapted isolate, AQ104, of African origin, able to adhere with different affinity to ICAM-1 and CD36 receptors as previously reported [36]. The adhesion assays were performed on the bone marrow-derived endothelial cell line HBMEC-60 [29] and the brain-derived endothelial cell line HBEC-5i [37], both acknowledged models of microvascular cells. Adhesion assays were conducted with and without a prior 12 h activation step with TNF-α a cytokine commonly used to simulate the inflammatory environment that typically characterizes development of cytoadherence [27]. In these experiments we tested the effect of CNF1 at a 10^−10^ M concentration, the dose at which the toxin is active in other *in vitro* and *in vivo* systems. The experiment included a pre-treatment assay, in which both endothelial cells were pre-incubated overnight with CNF1 before exposure to pRBCs, and a post-treatment assay, in which the toxin was incubated for 4 h after the pRBCs had adhered to endothelial cells. The results showed that pre-treatment with CNF1 compared to TNF-α activated controls decreased pRBC cytoadherence of both clones ItG and AQ104 to HBMEC-60 to 48% and to 51% (p<0.001) respectively, while using HBEC-5i model cytoadherence decreased up to 40% for ItG clone and 45% for AQ104 clone (Fig 1A). In the post-treatment experiment, results showed that CNF1 incubation induce a reduction in the number of adhering pRBCs up to a 40% and 42% (p<0.001) on HBMEC-60 monolayers and up to 48% for ItG clone and AQ104 respectively (Fig 1B). The adhesive behavior with respect to ICAM-1, was determined also using a purified recombinant ICAM-1 protein spotted on plastic for adhesion assay using both parasite lines, in order to verify whether CNF1 could affect parasites’ adhesion in absence of host signaling mechanism. Both ItG and AQ104 bound strongly to the purified protein ICAM-1, irrespectively of CNF1 addition, supporting the hypothesis that CNF1 does not directly interfere with host receptors (S2 Fig).

These experiments show a role of the Rho GTPases’ activator CNF1 [21] both in preventing pRBC adhesion and, more importantly, in reversing adherence of pRBCs to endothelial cells. This result is in a way in line with previous works on the effects on *P*. *falciparum* cytoadhesion of drugs, such as Fasudil and Atorvastatin [7,8]. In fact, although tested on *in vitro* cytoadherence models different from our models, both Fasudil and Atorvastatin are able to prevent cytoadherence, by acting on Rho GTPases-dependent pathways [7,8].

### CNF1 affects parasite cytoadherence by acting on endothelial cells

**Fig 2:**
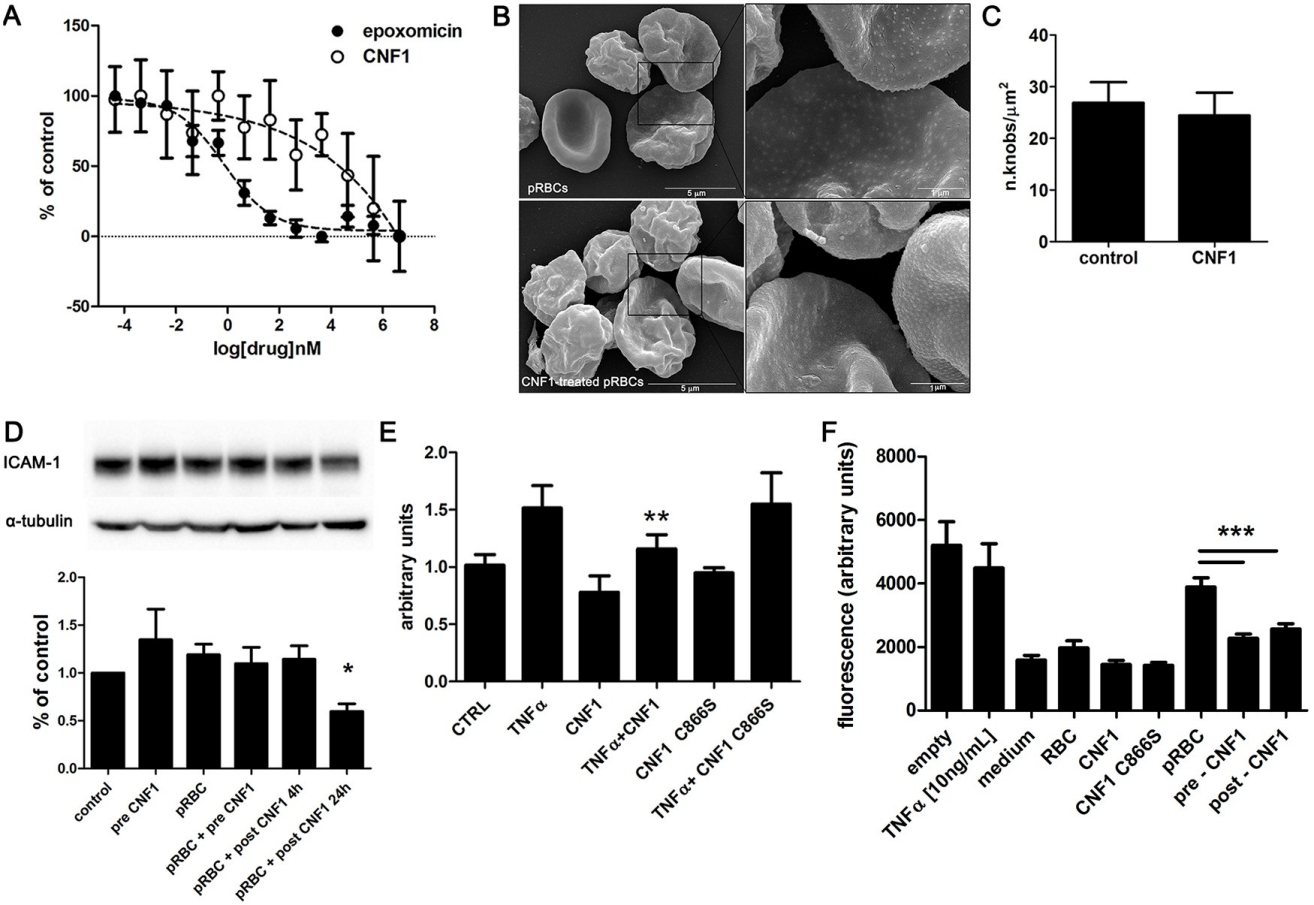
CNF1 affects the cytoadherence of pRBCs by acting on endothelial cells. (A) Dose response experiments for CNF1 effect on *P*. *falciparum* viability, assessed by pLDH enzymatic assay. *P*. *falciparum* asexual stages were treated with increasing concentrations of CNF1 and epoxomicin as reference drug. Data represent mean ± SEM from at least three experiments. (B) Scanning electron micrographs of pRBCs, untreated (upper panels) and treated with CNF1 (lower panels). Note the production, on the surface of infected erythrocytes, of regularly structured knobs that are not modified (higher magnification, right panels) by the challenge with CNF1. (C) Statistical analysis shows no differences in density of knobs per unit area (knobs/μm^2^) between CNF1-treated or untreated mature trophozoites (P = 0.89; n = 10). (D) CNF1 was added to HBEC-5i cells either overnight before the adhesion process (pre CNF1) or after the adhesion assay of pRBCs, for 4 or 24 h (post CNF1). Cells were lysed and processed for western blot analysis. The ICAM-1 immunoblot showing a representative experiment (upper panel) was normalized as a function of α-tubulin (lower panel) and expressed as arbitrary units (histogram). Note that CNF1 is able to down-regulate ICAM-1 only at 24 h of treatment. Data, expressed as percentage of control (= 1), represent mean ± SEM from at least three independent experiments. * p<0.05. (E) Flow cytometry analysis of ICAM-1 receptor by cultured HBEC-5i. Cells were exposed to TNF–α (10 ng/mL), CNF-1 alone and in combination for 12 hours. Histograms represent level of ICAM-1 expression as arbitrary units calculated by the formula: MFC (sample labeled with ICAM-1 antibody)/ MFC (sample labeled with IgG2a). Data represent mean ± SEM from at least three independent experiments. ** p<0.01. (F) Effect of CNF1 treatment on the integrity of the *P*. *falciparum*–exposed endothelial monolayer. HBEC-5i were grown on Transwell inserts to form a cell monolayer and were exposed for 2 h to, control RBCs, TNF–α (10 ng/mL), ItG-pRBCs before and after CNF1 treatment. Endothelial barrier integrity was assessed by means of passage of FITC-dextran dye into lower compartments and optical density (OD) analysis (630 nm). The OD of the dye was significantly lower in pRBC-exposed CNF1 pre- and post-treated endothelial monolayers than in pRBC-exposed untreated HBEC-5i cells. Note that CNF1 treatment can restore the integrity of the *P*. *falciparum*–exposed endothelial monolayer. Data represent mean ± SEM from at least three experiments *** p<0.001.

To understand which is the target of the mechanism enabling CNF1 to affect pRBC-adhesion to endothelial cells, we first asked whether this phenomenon was due to a cytotoxic effect of the toxin on the parasites. CNF1 showed no significant antiplasmodial activity, when compared to the reference active drug epoxomicin [30] (Fig 2A), indicating that the parasite viability was not impaired by the toxin.

Cytoadherence of pRBCs to endothelial cells is mediated by knobs, electron-dense, conical evaginations of the infected erythrocyte surface triggered by parasite infection [38]. As knob structure involves erythrocyte cytoskeleton modification, we analyzed whether the described ability of CNF1 to modify the host cell cytoskeleton had an impact on the pRBC knobs. ItG schizont-infected RBCs were purified and treated with CNF1 for 4 h, prior to be processed for SEM analysis. This showed that the morphology and concentration of knobs on the surface of CNF1-treated and untreated pRBCs appeared alike, indicating that CNF1 does not apparently prevent or reverse parasite cytoadhesion by affecting the density or the ultrastructure of knobs on the pRBC surface. Data obtained analyzing the density of knobs per unit area (knobs/mm^2^) (S1 Fig), confirm that CNF1 treatment of *P*. *falciparum* trophozoites did not affect the expression of surface knobs (Fig 2B and 2C), suggesting a knob-independent mechanism underlying CNF1 activity on cytoadherence.

As pRBC cytoadherence to the deep brain microvasculature involves interaction with human endothelial receptors, such as ICAM-1 [39], we analyzed the expression of this receptor in the brain-derived endothelial cells HBEC-5i. CNF1 was unable to counteract the TNF-α-induced increase of ICAM-1 (Fig 2D) in cells if added overnight before the adhesion process and also if added for 4 h after the parasites adhesion. A consistent decrease of ICAM-1 was observed only later, at 24 h of CNF1 challenge when the parasites detachment had already occurred. As expected, TNF-α treatment increases ICAM-1 expression levels compared to untreated cells. Treatments with CNF1 alone or combined with TNF-α decreased ICAM-1 expression. As regard treatments with the mutant CNF1 C866S, alone or after TNF-α stimulation, no significant changes in ICAM-1 expression were observed (Fig 2E). This suggests that CNF1 may revert cytoadherence also possibly favoring ICAM-1 internalization, in accordance to previous works that demonstrate that the toxin can induce endocytosis by acting on the Rho GTPases [40].

It is worth noting that beyond ICAM-1, also the cluster of differentiation 36 (CD36) protein and the endothelial protein C receptor (EPCR), are candidate receptors for the deadly complication of cerebral malaria [41]. Considering that the *P*. *falciparum* ItG line is unable to bind HBEC-5i by CD36, as these cells do not express CD36 [42], and that recent data have highlighted a discrepancy regarding the identification of EPCR as the major host receptor for pRBC sequestration in the brain during cerebral malaria [43], we focalized our attention on ICAM-1 receptor Also, it should be pointed out that this study deals only with a specific *in vitro* CM model that implies the known relationship between CNF1 and ICAM-1 receptors [44]. Thus, in order to highlight CNF1 pharmacological potentiality on cytoadherence, further studies will be necessary to understand the interaction between CNF1 and the other host receptors involved in CM.

To explore additional mechanisms, we analyzed if CNF1 could influence signaling pathways affecting endothelial barrier permeabilization. Maintenance of the blood–brain barrier integrity [37] is relevant to tightly control molecular and cellular trafficking into the brain [45,46]. In CM, endothelial hyperpermeability and high plasma levels of proteins, such as homocysteine that leads to inflammation in the blood vessels, are observed [47] and the latter could lead to a prothrombic state [48]. In addition, the endothelium can be damaged by the action of polymorphonuclear cells and monocytes, whose presence is associated to *P*. *falciparum* disease severity [49,50]. Interestingly, hyperpermeability and microvascular leakage, both involved in the pathology of CM, have been reported to be mediated by Rho signaling [51]. Experiments to investigate possible effects of CNF1 on endothelial permeability were performed on the brain derived HBEC-5i cell line. Cells were cultured to confluence on Transwell inserts and exposed for 2 h to ItG pRBCs (schizont stages). Endothelial monolayer integrity was determined by measuring levels of FITC-dextran dye passing through the cell layer to the lower compartment. TNFα stimulation was used as a positive control for *in vitro* endothelium disruption. The experiment showed that HBEC-5i cell layer permeability was significantly higher in the presence of pRBC (p<0.001) compared to incubation with uninfected RBCs (Fig 2F). It also showed that pRBC-induced permeability of the HBEC-5i cell layer was prevented by incubation of CNF1 before the pRBC were incubated with endothelial cells and could be reversed by incubating CNF1 on the HBEC5i cells with adhered parasites. These results are consistent with the notion that some of the pathways targeted by CNF1, including Rho, Rac and Cdc42 as well cAMP signaling [52], play a key role in controlling the endothelial permeability [53,54]. Cdc42 activation, in particular, has been demonstrated to restore the endothelial barrier integrity [55].

Altogether, these experiments indicate that CNF1 prevents and reverts pRBC cytoadherence mainly, and probably entirely, acting on endothelial cells. The hypothesis of a crucial role played by the Rho GTPses in reinstate the endothelia strength is in accordance with the herein demonstrated influence of CNF1 on cytoadherence that is also strictly dependent on Rho GTPases activity.

### CNF1 both prevents and reverses pRBC cytoadherence via activation of the Rho GTPases in endothelial cells

**Fig 3:**
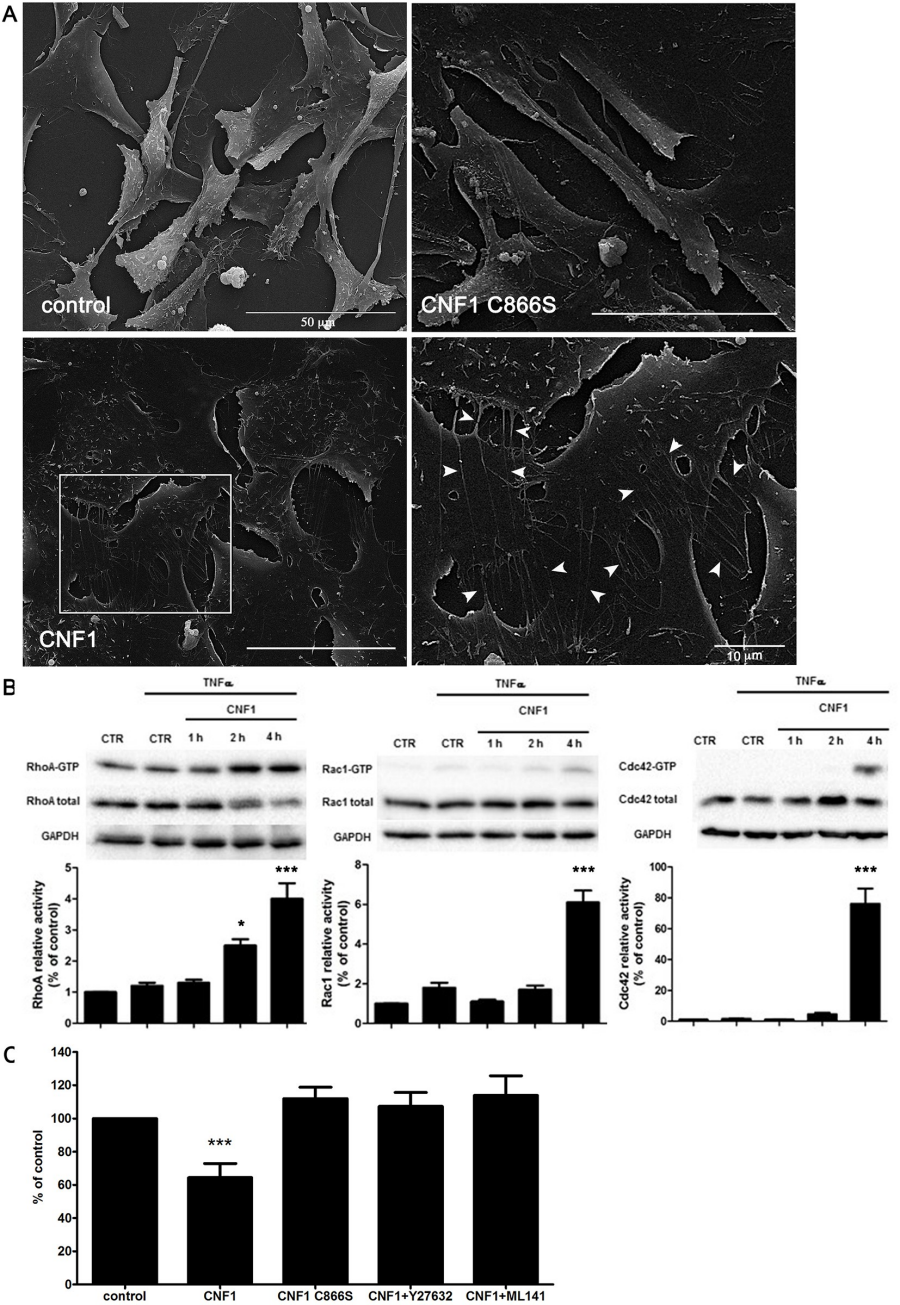
CNF1-induced detachment of pRBCs from endothelial cells is crucially dependent on the concomitant activation of the Rho GTPases. (A) SEM analysis showing HBEC-5i cells activated with TNF-α (control) and TNF-α-activated cells treated with CNF1 or with CNF1 C866S. Note that, whereas cells exposed to the mutant toxin display a surface morphology very similar to that of control cells, CNF1 promotes an evident spreading and flattening of cells, increasing the monolayer confluence. Also, philopodia-like structures, clearly visible both on the cell surface and between cells are induced by the toxin (inset in c, arrowheads). (B) Activation kinetic of RhoA, Rac1 and Cdc42 GTPase. HBEC-5i cells were activated with TNF-α overnight and then treated with CNF1. At the indicated time points, cells were lysed and the active form of RhoA, Rac1 and Cdc42 GTPases present in each sample was detected by pull-down assays, as described in Materials and methods. Immunoblots showing a representative pull-down experiment (upper panels) were normalized as a function of the total RhoA, Rac1 or Cdc42 proteins loaded in the assay and expressed as relative activity (below panels). Note that CNF1 activated Rho GTPases starting from 4 h of treatment. Data, expressed as percentage of control (= 1), represent mean ± SEM from at least three independent experiments. * p<0.05 compared to control; *** p<0.001 compared to control (C) Reversal effect on ItG pRBC binding to TNF-stimulated endothelial cells HBEC-5i after CNF1 post-treatment assays performed in the presence of the mutant CNF1 C866S, the RhoA inhibitor Y27632 and the Rac1/Cdc42 inhibitor ML141. pRBC bound were expressed as a percentage (%) bound pRBC.mm-2 compared to control (PBS only). Note that the CNF1 C866S treatment does not cause the pRBC detachment, while the inhibition of RhoA, or of Rac1/Cdc42, impairs the ability of CNF1 to detach pRBCs from endotelial cells. Data are expressed as the mean percentage of control ± SEM from at least three experiments. ± SEM, *** p<0.001.

Therefore, we investigated the morphological and biochemical changes induced by CNF1 in endothelial cells. Scanning electron micrographs (Fig 3A) show that exposure to the toxin caused flattening and spreading of brain endothelial cell monolayers, increased the number of philopodia-like structures extending from the cell surface and displayed only a minor ruffling activity. In contrast, cells exposed to the mutated recombinant toxin (CNF1 C866S), in which the serine substitution at position 866 abrogates the enzymatic activity on Rho GTPases [21], show the same surface morphology of untreated cells. It is worth noting that cell viability was not affected when cells were treated with CNF1 or CNF1 C866S at different dilutions (from 1.25x10^-8^ M to 10^−10^ M, this last being the working dilution used in our experiments (S3 Fig).

As this indicates that the observed cytoskeleton-dependent morphological changes are under the control of proteins members of the family of Rho GTPases, we performed pull-down assays on CNF1-treated cells to verify the activation state of these regulatory proteins at 4 h, the incubation time used for cytoadherence studies. In particular, the relative activity of RhoA, Rac1 and Cdc42 GTPases (proteins belonging to the Rho, Rac and Cdc42 subfamilies, respectively) in CNF1-treated samples was normalized with respect to the activity of the same GTPase in control cells (= 1). The graphs reported in Fig 3B show that activated RhoA, Rac1 and Cdc42 were all increased after 4 h of CNF1 challenge, although at a different extent. Indeed, while the fold-increase of RhoA and Rac1 GTPases at 4 h was only 4 and 6.4, respectively, Cdc42 GTPase was strongly activated by CNF1 (76 fold-increase). As expected, no activation of RhoA, Rac1, and Cdc42 was induced either by the recombinant CNF1 C866S or by the heat-inactivated CNF1 (S4 Fig).

To evaluate the role played by the CNF1-activated Rho GTPases in both prevention and reversion of cytoadherence (Fig 3C), we first used the mutant toxin CNF1 C866S, whose inability to affect cytoadherence clearly indicates the need of the Rho GTPase pathway in both prevention and rescue of pRBC adhesion to endothelial cells. However, although representing an invaluable tool to study the role of the Rho GTPases in all CNF1-induced effects, yet CNF1 C866S does not permit to identify which of the Rho GTPase subfamilies is involved in a particular response. Therefore, to address this question, we have used the inhibitor Y27632, a cell-permeable, reversible, inhibitor of Rho kinases (ROCK). Also, we have employed a potent, selective and reversible non-competitive inhibitor of Rac/Cdc42, ML141, either in the pre-treatment or in the post-treatment binding assay. In both cases, inhibitors were added to endothelial cells either 1 h before CNF1 treatment or together with CNF1. The experiments showed that the number of attached parasites was decreased, as expected, by both pre- and post- treatment with CNF1, whereas their number was not affected in either the pre-incubation and in the co-incubation of Y27632 or ML141 with CNF1.

### The CNF1-induced changes in the actin cytoskeleton organization require the activation of Rho, Rac and Cdc42

**Fig 4:**
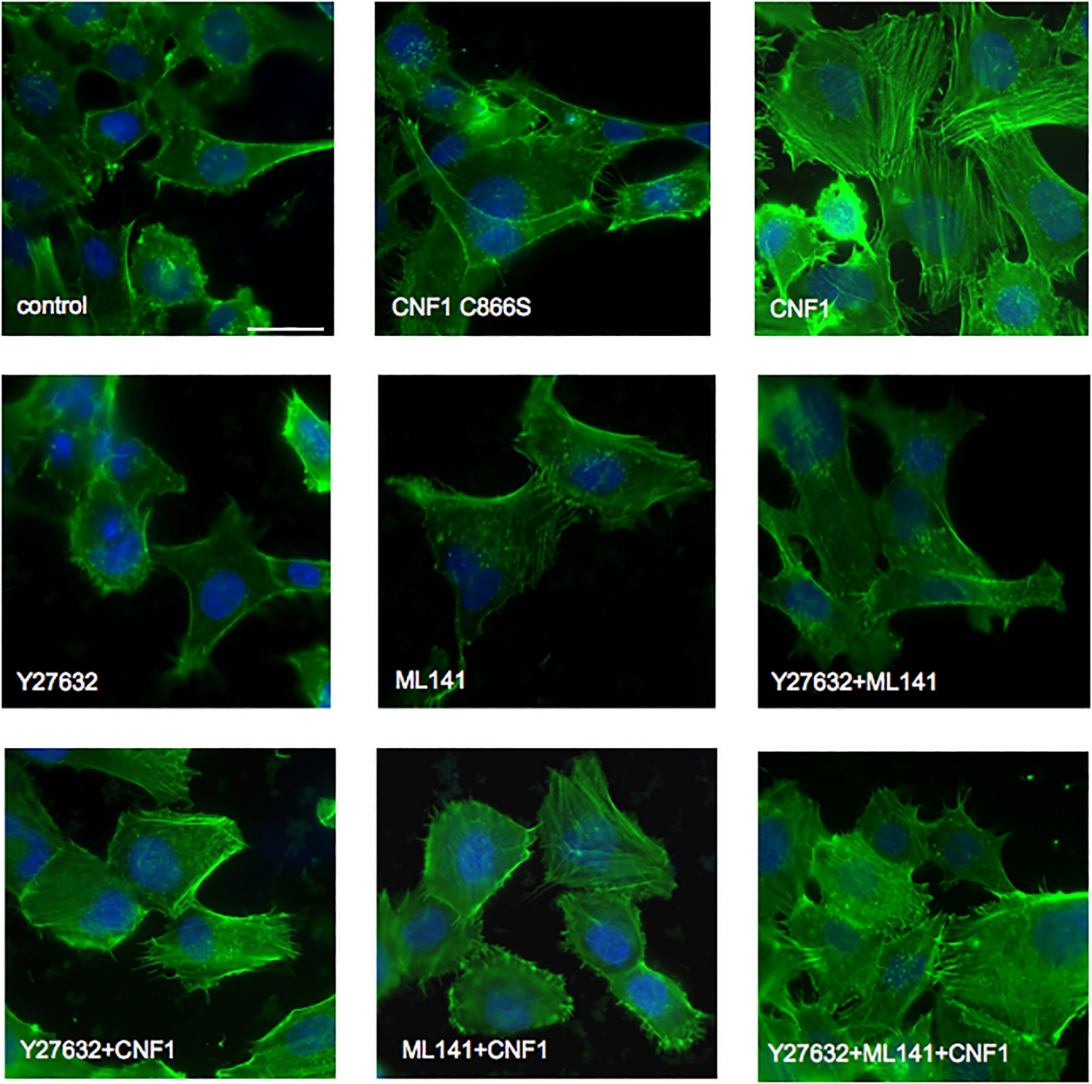
CNF1 reorganizes actin cytoskeleton in endothelial cells. Fluorescence micrographs of HBEC-5i cells stained with FITC phalloidin for F-actin detection. Note that CNF1, but not CNF1 C866S, induce a remarkable reorganization of actin in stress fibers and membrane protrusions and that these modifications are partially reduced when cells are pre-treated with the Rock inhibitor Y27632 and the Rac/Cdc42 inhibitor ML141. Bar = 10 μm.

Since one of the main target of the Rho GTPases’ activity is the actin cytoskeleton and CNF1 is largely proved to affect the cytoskeletal dynamics [56], we performed experiments to visualize how the above reported treatments with toxins and inhibitors could influence the F-actin distribution in endothelial cells (Fig 4). As expected, CNF1 challenge promotes a remarkable reorganization of the actin cytoskeleton, which appears enriched and well organized in stress fibers and membrane protrusions. This effect was confirmed by a semi-quantitative analysis of phalloidin signal intensity (S5 Fig). By contrast, control cells, cells treated with CNF1 C866S, and cells exposed to the Rock (Y27632) and Rac/Cdc42 (ML141) inhibitors, either alone or in combination, show a poorly organized actin network Interestingly, the cell pre-treatment with the above inhibitors impaired the typical ability of CNF1 to reshape the actin cytoskeleton (Fig 4, bottom line).

Taken altogether, our results clearly show that, by acting on the Rho GTPases, CNF1 prevents cytoadherence to endothelial cells and, more importantly, induces the detachment of infected erythrocytes. These phenomena, which are accompanied by changes in the endothelial cell surface with ICAM-1 downregulation, are sensitive to inhibitors of Rho and Rac/Cdc42 activity. Our hypothesis is that the unrivaled ability of CNF1 to simultaneously activate the Rho GTPases’ subfamilies, which are antagonistic under normal conditions [34,57–60], could trigger a signal that in turn activates a cascade of events affecting cytoadherence, possibly including ICAM-1 receptor internalization. Our data clearly show that the detachment may occur only if both Rho and Rac/Cdc42 subfamilies are active in the same time frame. It is conceivable that the observed phenomenon of continued adherence by non-viable *P*. *falciparum* pRBCs [32] may be targeted by adjuvant therapies designed to prevent or preferably reverse cytoadherence. Furthermore, it is important to underline that pharmacologically targeting endothelial cells and not the parasites prevents insurgence of parasite drug resistance. Hence, by acting on endothelial cells, CNF1 could reverse cytoadherence, thus unblocking microvascular flow. In addition, it should be underlined that the main cause of coma in CM is unknown, and besides obstruction, a range of other possible contributory mechanisms, like the cytokines activation, has been postulated, thus rendering CM a multifactorial diseases [61]. Therefore, the CNF1 ability to counteract neuroinflammation *in vivo* [62] could make this protein even more attractive as a prospective life-saving single dose-drug for fatal complications due to CM, also because CNF1 appears to be effective within a few hours from the start of treatment. CNF1 treatment may also represent an adjunctive therapy to be used in association with other validated antimalarial agents. Further and more detailed studies on other pathological phenotypes will be necessary in order to evaluate the real CNF1 impact against CM.

## Supporting information

S1 Fig (A) A selected slightly flat area of a mature trophozoite with knobs; (B) IMAGE J elaboration of the same area with an adjusted signal threshold; (C) analyzed particles showed in B. (TIF)

S2 Fig Adhesion of ItG and AQ104 pRBC per mm^2^ to purified ICAM-1. Different concentrations of recombinant ICAM-1 protein were spotted on plastic and exposed to pRBC for 1 h in presence or absence of CNF1.
(TIF)

S3 Fig Cell viability, determined by NRU assay following treatment with different concentration of CNF1 and the mutant CNFC866S for 4 or 24 h, was expressed as percentage compared to control. Results are expressed as mean ± S.E.M. from three separate experiments performed in triplicate.
(TIF)

S4 Fig Immunoblots showing representative pull-down experiments of RhoA, Rac1 and Cdc42 GTPase in control HBEC-5i cells and in cells exposed to heat-inactivated (Hi) CNF1 and to the mutant CNF1 (CNF1 C866S), for 4 h. Note that neither Hi-CNF1 nor CNF1 C866S are able to activate Rho GTPases.
(TIF)

S5 Fig Phalloidin immunofluorescence intensity was analyzed by IMAGE J [33], in order to quantify the F-actin polimeryzation induced by CNF1 on endothelial cells. Quantification of phalloidin intensity signal derives from the means of grayscale intensity value for each image. Results are expressed as mean ± S.E.M. from five images for each sample (n = 5), acquired at the same magnification, fluorescence exciting and gain conditions.
(TIF)
