# Supplementary figures and images for: The bacterial protein CNF1 as a new strategy against Plasmodium falciparum cytoadherence
Source: PLoS One. 2019 Mar 7;14(3):e0213529. doi: 10.1371/journal.pone.0213529 (PMC6405130; doi:10.1371/journal.pone.0213529)

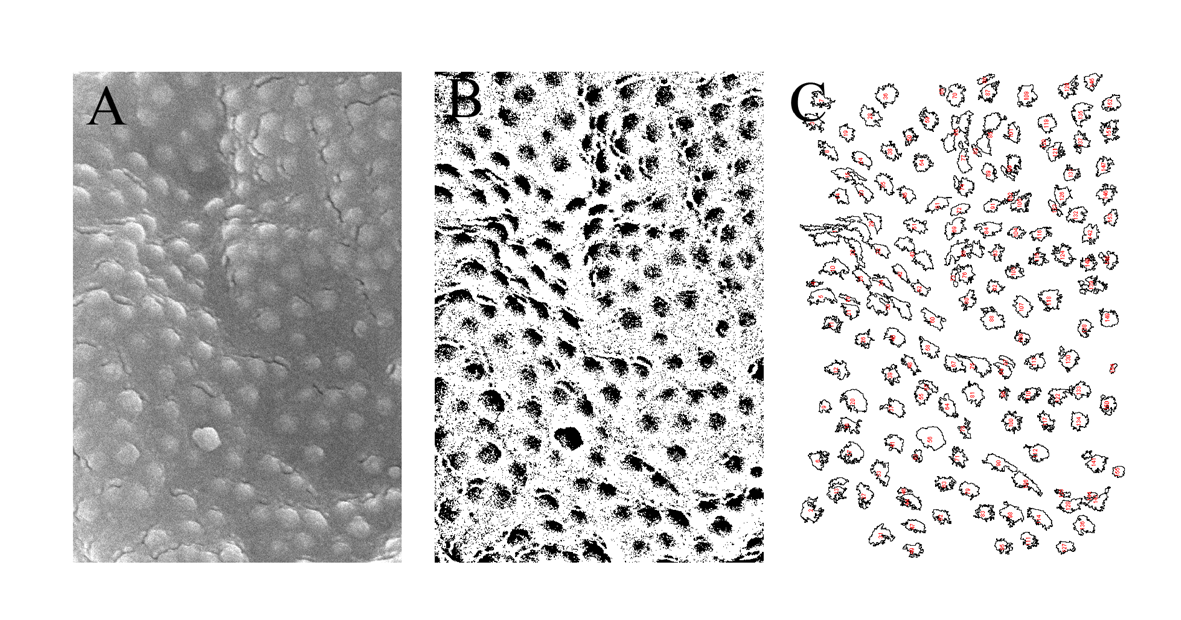

Supplement: S1 Fig — (TIF) [file pone.0213529.s001.tif]

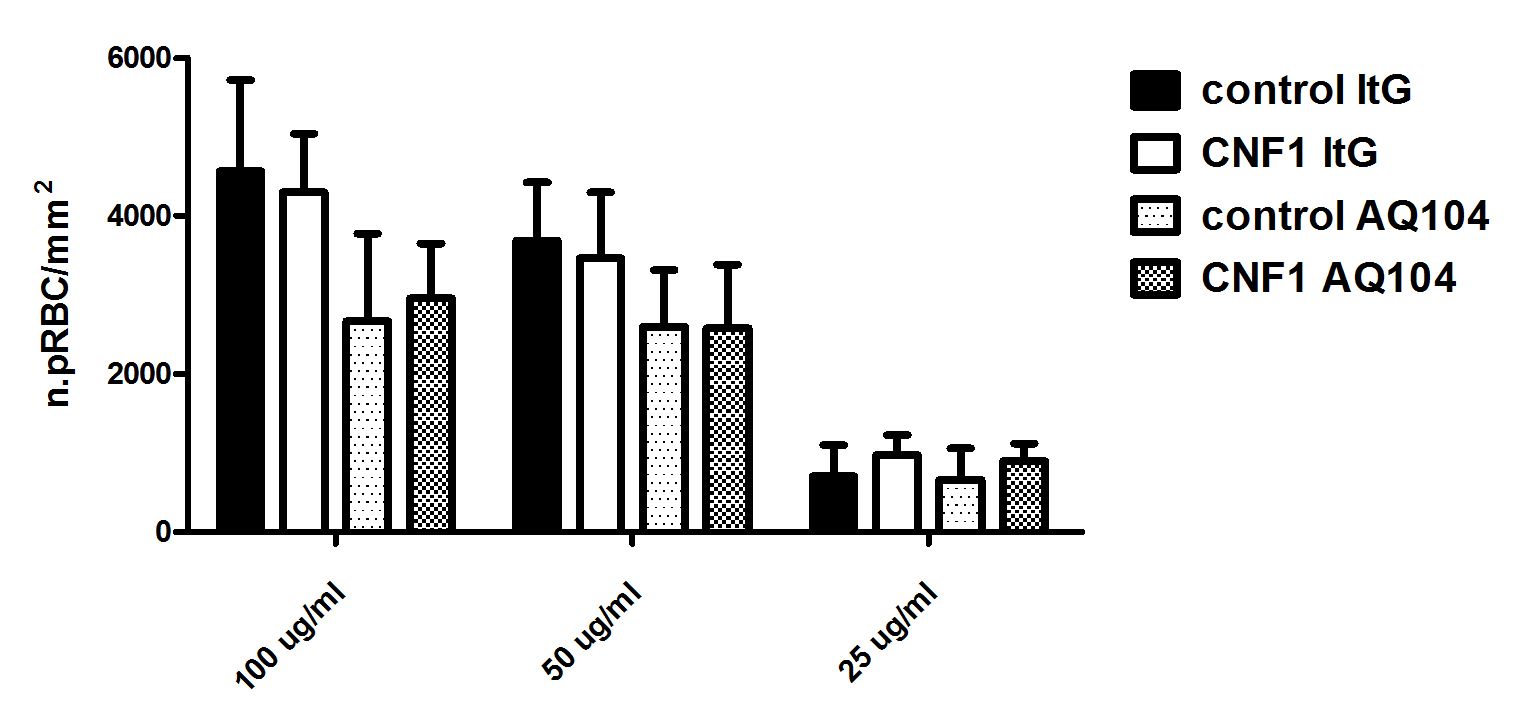

Supplement: S2 Fig — Different concentrations of recombinant ICAM-1 protein were spotted on plastic and exposed to pRBC for 1 h in presence or absence of CNF1. (TIF) [file pone.0213529.s002.tif]

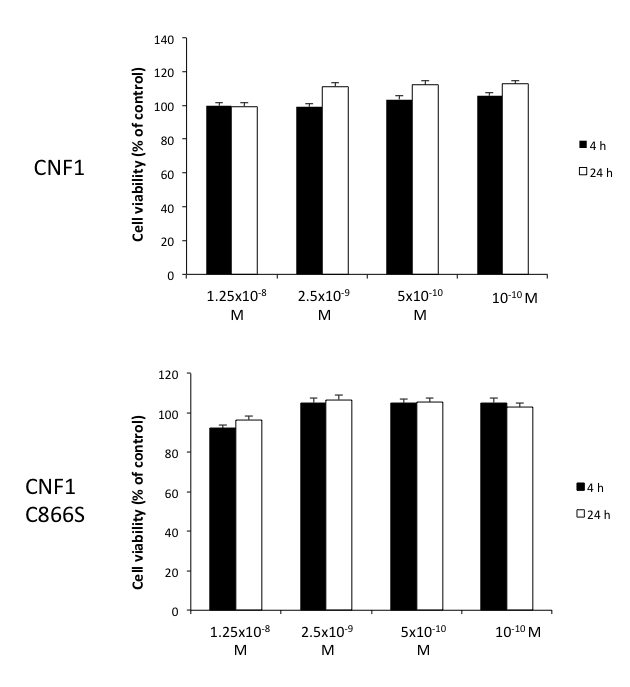

Supplement: S3 Fig — Results are expressed as mean ± S.E.M. from three separate experiments performed in triplicate. (TIF) [file pone.0213529.s003.tif]

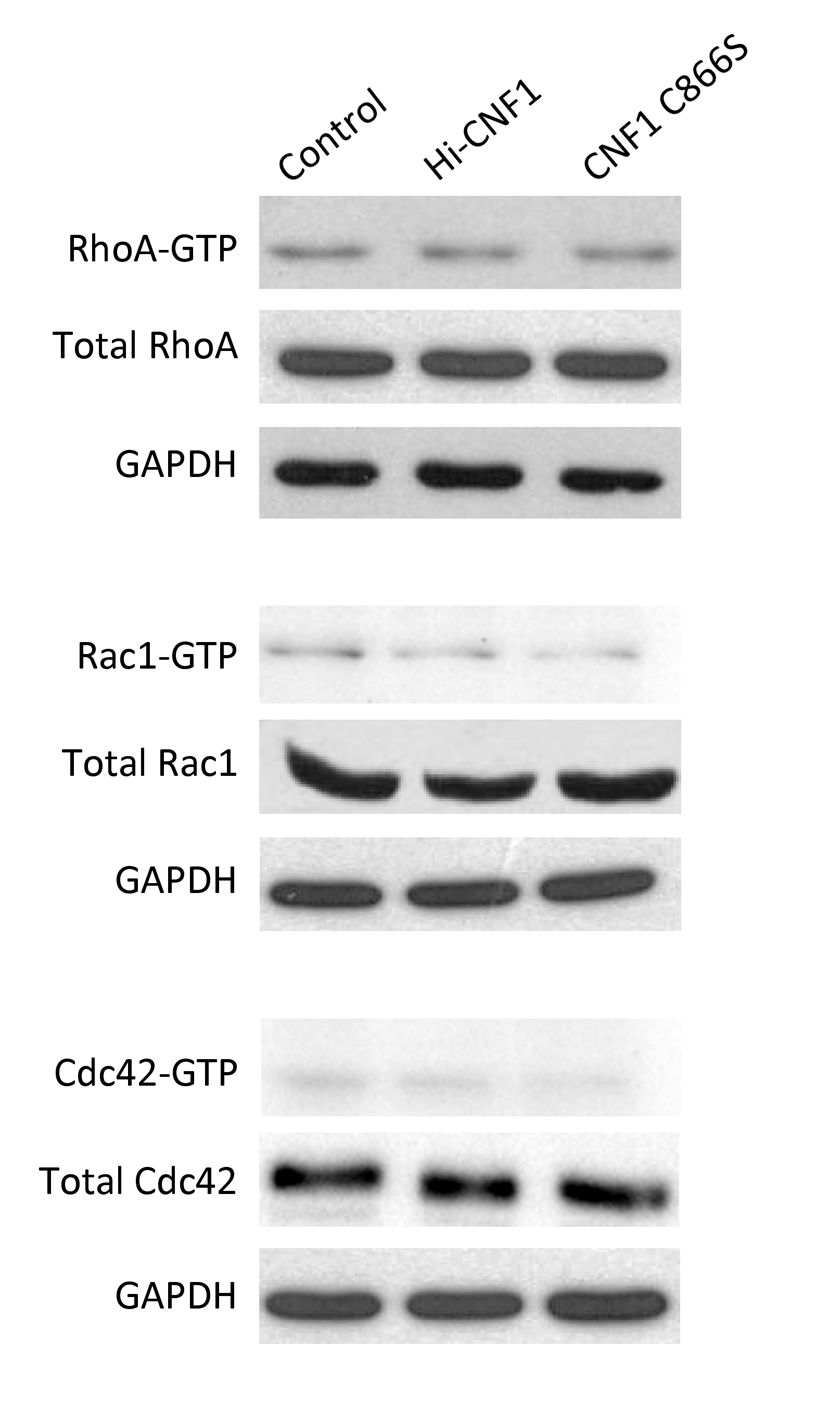

Supplement: S4 Fig — Note that neither Hi-CNF1 nor CNF1 C866S are able to activate Rho GTPases. (TIF) [file pone.0213529.s004.tif]

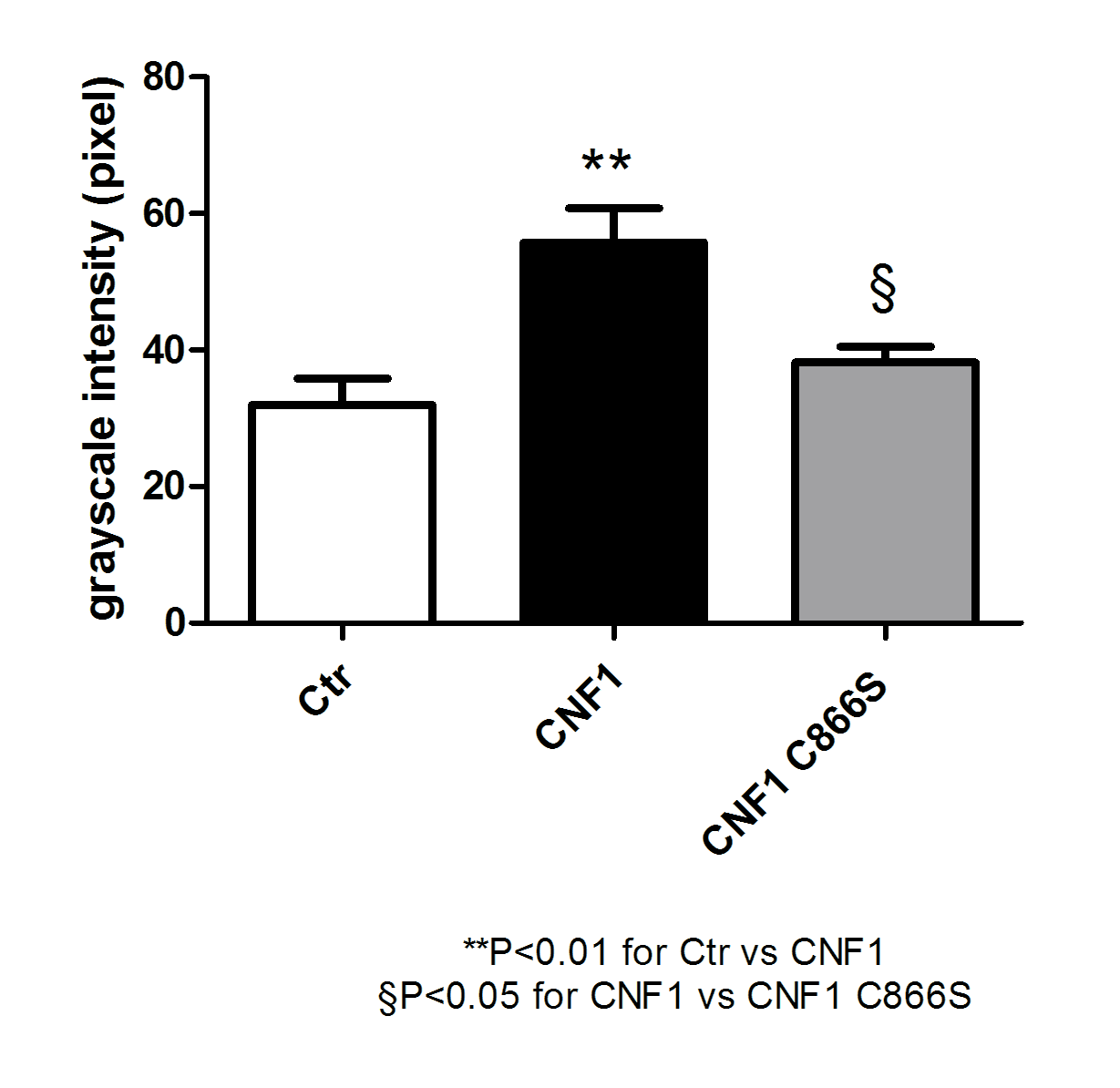

Supplement: S5 Fig — Quantification of phalloidin intensity signal derives from the means of grayscale intensity value for each image. Results are expressed as mean ± S.E.M. from five images for each sample (n = 5), acquired at the same magnification, fluorescence exciting and gain conditions. (TIF) [file pone.0213529.s005.tif]
